# Biologically engineered microbes for bioremediation of electronic waste: Wayposts, challenges and future directions

**DOI:** 10.1049/enb2.12020

**Published:** 2022-02-26

**Authors:** Ping Han, Wei Zhe Teo, Wen Shan Yew

**Affiliations:** ^1^ Synthetic Biology for Clinical and Technological Innovation National University of Singapore Singapore Singapore; ^2^ Synthetic Biology Translational Research Programme Yong Loo Lin School of Medicine National University of Singapore Singapore Singapore; ^3^ Department of Biochemistry Yong Loo Lin School of Medicine National University of Singapore Singapore Singapore

**Keywords:** biochemical engineering, bio‐economy, biological design, microbial engineering, synthetic biology

## Abstract

In the face of a burgeoning stream of e‐waste globally, e‐waste recycling becomes increasingly imperative, not only to mitigate the environmental and health risks it poses but also as an urban mining strategy for resource recovery of precious metals, rare Earth elements, and even plastics. As part of the continual efforts to develop greener alternatives to conventional approaches of e‐waste recycling, biologically assisted degradation of e‐waste offers a promising recourse by capitalising on certain microorganisms' innate ability to interact with metals or degrade plastics. By harnessing emerging genetic tools in synthetic biology, the evolution of novel or enhanced capabilities needed to advance bioremediation and resource recovery could be potentially accelerated by improving enzyme catalytic abilities, modifying substrate specificities, and increasing toxicity tolerance. Yet, the management of e‐waste presents formidable challenges due to its massive volume, high component complexity, and associated toxicity. Several limitations will need to be addressed before nascent laboratory‐scale achievements in bioremediation can be translated to viable industrial applications. Nonetheless, vested groups, involving both start‐up and established companies, have taken visionary steps towards deploying microbes for commercial implementation in e‐waste recycling.

## 1 INTRODUCTION

Remarkable advances in science, engineering, and technology since the early 20th century have created amazing tools and devices that have revolutionised our world and become arguably indispensable in our daily lives. In many developed countries, higher levels of disposable incomes and growing urbanisation have driven a fervent pace of technological change and the adoption of electrical and electronic gadgets. While modern technology has undeniably elevated living standards and ushered in lifestyle conveniences, it has, at the same time, left in its wake two distressing impacts on the environment—pollution and depletion of natural resources.

**FIGURE 1:**
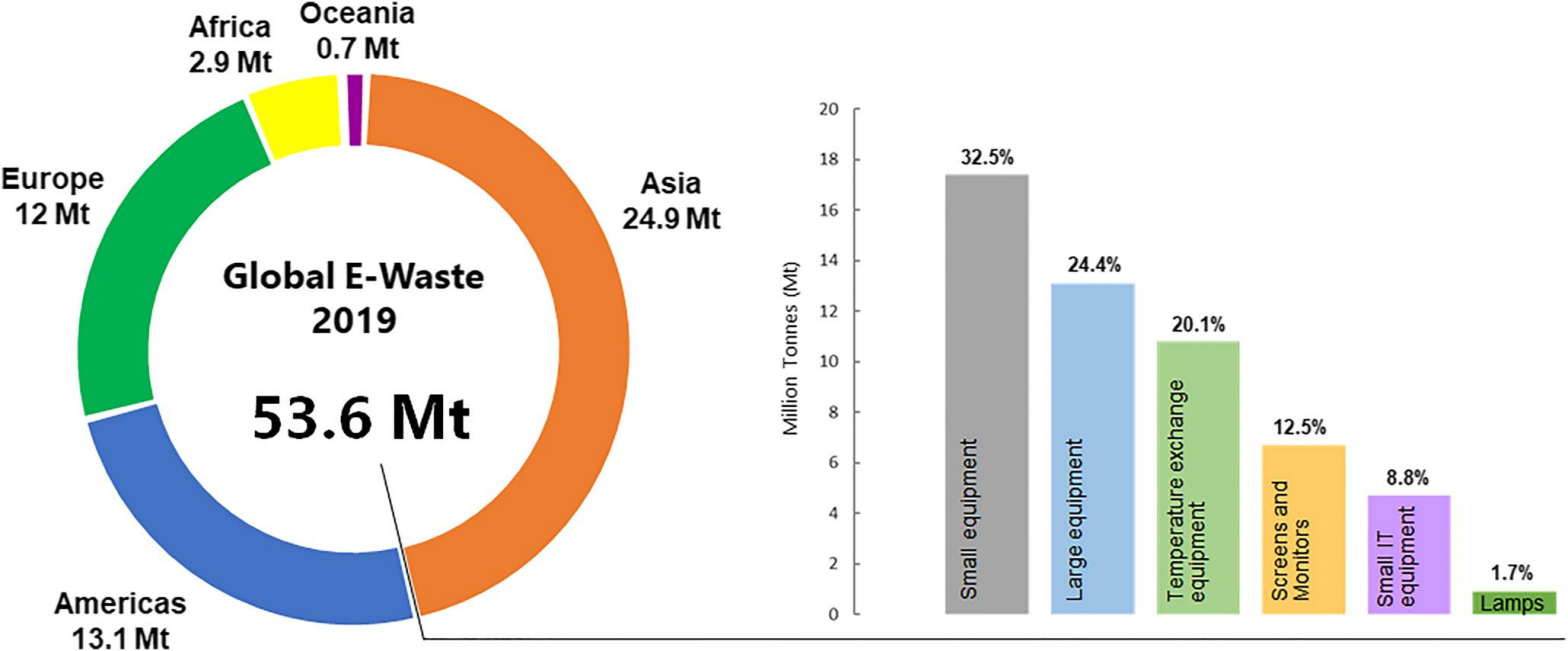
Global e‐waste generated in 2019. Regionally, Asia generated the largest volume of e‐waste and Oceania, the least. In 2019, the global quantity of e‐waste mainly comprised small equipment (17.4 Mt), large equipment (13.1 Mt), temperature exchange equipment (10.8 Mt), screens and monitors (6.7 Mt), small IT and telecommunication equipment (4.7 Mt) and lamps (0.9 Mt). Of these, 9.3 Mt, or 17.6%, was documented to be collected and properly recycled, while the fate of 82.6% remained uncertain. Source: Global E‐Waste Monitor 2020

In 2019, the Global E‐waste Monitor 2020 reported a record 53.6 million metric tons of electrical and electronic waste (e‐waste) generated, with small electronic devices representing the largest proportion of e‐waste, followed by large equipment and consumer appliances (Figure 1). [1] Although this volume is modest compared to the 242 million tonnes of plastic waste, which constituted the 2.01 billion tonnes of municipal solid waste the world produced in 2016, e‐waste presents one of the fastest growing waste streams, with an estimated growth rate of 3%–5% annually. [2, 3] It was predicted that by 2030, that global e‐waste will increase up to 74 million tonnes, impelled by a burgeoning penetration of electric and electronic equipment in developing countries, a foreseeable replacement market in developed countries and increasing product obsolescence rates. [1]

**FIGURE 2:**
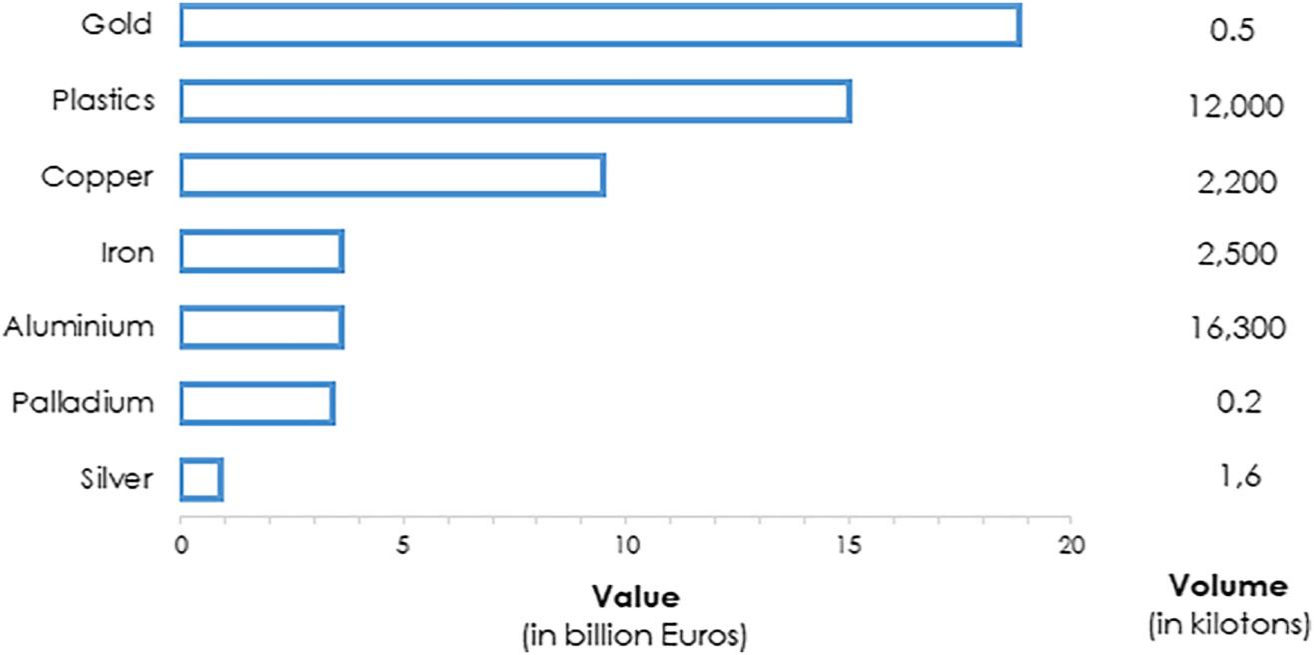
Potential value and volume of raw materials in e‐waste worldwide in 2016. E‐waste contains not only precious metals including gold, silver, copper, platinum, and palladium but also valuable bulky materials such as iron and aluminium and plastics that are recyclable. Secondary raw materials of e‐waste are estimated to be worth 55 Billion Euros. Source: Global E‐Waste Monitor 2017

Composed of diverse metals and non‐metals, the constituents of e‐waste differ across product lines and categories and can contain, in addition to prevailing base metals and varying quantities of plastics and ceramics, both precious metals and rare Earth elements. [2, 4] Waste electrical and electronic equipment (WEEE), however, also often contain hazardous materials including heavy metals such as lead, nickel, chromium, and Mercury, and persistent organic pollutants (POPs) such as polychlorinated biphenyls (PCBs) and brominated flame retardants (BFRs). [5, 6] When improperly disposed of, these substances can either be directly released or act as a precursor for the generation of toxic byproducts, resulting in environmental pollution and severe health risks. [7, 8] Indeed, the management of e‐waste presents formidable challenges due to its massive volume, high component complexity, and associated toxicity. Yet, with as many as 69 elements from the periodic table found in complex electronics including precious metals such as gold, platinum, palladium, ruthenium, and silver, e‐waste presents an alternative or secondary source of raw materials in a circular economy (Figure 2). [4, 9] However, despite the potential for resource recovery, only 17.4% of e‐waste was formally collected and recycled globally in 2019, inferring a loss of high‐value commodities conservatively valued at $57 billion through disposal. [1] In the face of insatiable global demand and depleting geologic stores of extractable raw materials, urban mining—the extraction and purification of precious metals from waste streams—is increasingly being recognised to play a strategic role in resource security. It is argued that a shift to resource recovery through e‐waste recycling is becoming more cost‐effective compared to conventional virgin mining and that the grade and concentration of metals in e‐waste can be higher than that extracted from ores. [10].

Concomitant with the shifting mindset on the importance of e‐waste management is the impetus to explore greener approaches to e‐waste recycling compared to conventional chemical methods. In this review, research developments in the biologically assisted degradation of e‐waste is presented. While the maturing field of bioleaching has fostered the inception of nascent industry players that tap into the use of microorganisms for metal recovery, limitations of the bioleaching process are described. Ensuing are discussions on the potential of biological engineering of microorganisms to further advance the bioremediation of e‐waste not only for metal recovery but also for plastic recycling. Next, the technical challenges that need to be overcome in order to translate the technology into industrial implementation are addressed. Finally, open issues and the future prospects of e‐waste recycling using biologically modified microorganisms are outlined in the last part of the review.

## 2 BIOTECHNOLOGY: A GREENER APPROACH TO E‐WASTE RECYCLING

E‐waste recycling encompasses the collection of WEEE, its processing, and finally the recovery of valuable materials. In current industrial practices, the initial stage involves dismantling and granulation of WEEE using physical–mechanical methods and separation of recyclable fractions from the non‐essential components via density, magnetic, or current‐based sorting techniques. [11, 12] Plastics, which account for almost 20% of e‐waste, are mostly given a second lease of utility as recycled polymers, although emerging technologies aim to transform e‐waste plastics into higher value products through microfactories. [13] For the majority of industrial scale e‐waste recycling, metals are recovered using pyrometallurgical and hydrometallurgical processes; the former involves thermal treatment, smelting in furnaces or alkali chemicals, and other solid‐liquid‐gas reactions at high temperature, while the latter uses solvent extraction, leaching, adsorption, and ion exchange processes. [14] Unfortunately, such traditional methods of metal reclamation from e‐waste tend to use, or release as part of the extraction process, toxic chemicals such as highly acidic/alkaline reagents, cyanide, and dioxins that are detrimental to human and ecological health. As part of efforts to minimise the emission of hazardous pollutants during e‐waste recycling, advanced technologies, such as electrochemical, supercritical, and vacuum metallurgical technology, as well as novel ones like ultrasound technology, are being developed often in combination with improved conventional methods. [15].

In the exploration of greener alternatives to conventional approaches, biologically assisted degradation of e‐waste offers a promising way to metal recovery. Microbe‐metal interaction is a naturally occurring phenomenon in which microorganisms, both prokaryotic and eukaryotic, acquire metal species from the external environment that are essential for various structural or catalytic functions. [16] A diverse group of microorganisms that includes heterotrophic and chemolithotrophic bacteria and fungi have an innate ability to convert insoluble metals to their soluble and extractable form—either by immediate surface attachment and directly oxidising minerals and solubilising the metals, or through an indirect action of regenerating of ferric ions, which serve as oxidising agents. [17, 18] This process of bioleaching, also known as biohydrometallurgy, involves various mechanisms such as complexolysis, acidolysis, redoxolysis, and bioaccumulation. [19].

In the initial phase, potential candidates for biorecovery of metals are often prospected from microbial communities inhabiting natural environmental niches or indigenous to polluted sites or operation facilities. As comprehensively reviewed by Valix [20] and Islam et al. [21], several microorganisms including bacteria (e.g. *Acidithiobacillus* spp., *Sulfobacillus* spp., *Pseudomonas* spp.) and fungi (e.g. *Aspergillus* spp., *Purpureocillium lilacinum*) have been reported for recovery of base (Cu, Fe, Ni, Pb, Zn) and precious metals (Ag, Au, Co, Pd, Pt) from e‐waste. In particular, the acidophilic group of bacteria plays a prominent role in bioleaching in e‐waste due to their lenience towards heavy metals. [22, 23].

Among WEEE, printed circuit boards (PCBs) are the predominant targets for recycling because of their valuable metals, which account for more than 95% of the recovery value. As integral components in innumerable electronic devices including the burgeoning end‐of‐life mobile phones, PCBs are composed of around 28%–30% metal, with up to 20% copper and precious metals such as silver, gold, and platinum making up 0.3%–0.4%. [24] Yet, despite the many studies, the commercial application of bioleaching of PCBs remains elusive for the reasons of low leaching efficiency and long leaching time. Besides component heterogeneity, the toxicity of the PCBs, which can affect the growth of microorganisms as well as interference by exogenous metals, have been thought to be contributing factors to low efficiency in bioleaching.

Research efforts have hence been increasingly directed towards improving bioleaching processes. One of the widely deployed methods to improve cell tolerance against e‐waste toxicity is the preadaptation of microorganisms through acclimatisation to increasing PCBs concentrations prior to bioleaching. [25, 26, 27, 28] A two‐step process whereby microorganisms are allowed to grow before exposure to the target waste was also found to be crucial in mitigating the inhibitory effects and additional extension to three‐ or four‐steps positively affected metal recovery. [28, 29, 30] In a further move to decouple cell viability from bioleaching activity, an indirect, non‐contact mechanism was investigated using bacteria‐free cultural supernatant. [31, 32].

The complex compositions of e‐waste make it challenging for single microorganisms to completely utilise the substrates and efficiently recover multiple metals in one process. As such, the use of mixed cultures or the development of a microbial consortium is gaining traction. [20, 33] Bioleaching of base metals requires different microbial strains from those for precious metals. Thus, using co‐ or mixed‐cultures can potentially harness synergy between groups of microorganisms not only in accessing nutrients but also in functional robustness. For example, copper and gold recovery efficiencies reached 98% and 44%, respectively, when copper leaching was carried out using chemolithotrophic acidophilic *Acidithiobacillus ferrivorans* and *Acidithiobacillus thiooxidans* followed by the use of cyanide‐producing heterotrophic *Pseudomonas fluorescens* and *Pseudomonas putida* for gold leaching. [34] And chromium recovery was reported to improve when co‐cultures of biosurfactant‐producing bacteria and sulphur‐oxidising bacteria were used. [35].

Besides biotic determinants to the bioleaching processes, several abiotic factors, such as initial pH, carbon source, pulp density, and particle size, and their interaction can also influence bioleaching efficiency. To maximise metal recovery, these parameters can be optimised under a multi‐objective strategy using response surface methodology in order to determine optimal bioleaching conditions. [36, 37] Bioleaching offers a promising route in the recovery of the metallic fractions from e‐waste and is considered more environmentally benign than physicochemical approaches. In the light of its importance, it is critical that the processes involved are optimised to attain levels of recovery efficiencies that enable the translation of research to sustainable, commercial application.

## 3 BIOLEACHING: FROM EXPLORATION TO IMPLEMENTATION

Fostered by years of research and development, microbial leaching has been successfully applied at industrial scale biomining operations for extraction of base and precious metals from sulfidic ores. [38] Biohydrometallurgical extraction of metals such as cobalt and nickel has been technically established and have progressed into commercial implementation. Today, metal recovery from low‐grade copper ores and refractory gold ores are the two dominant industrial applications and account for about 20% and 5% of global production of copper and gold, respectively. [33, 39, 40, 41] Although the use of microorganisms in metal recovery have found profitable actualisation in biomining, their application in the recycling of waste materials such as e‐waste is still emerging and remains largely confined to laboratory‐scale studies.

Despite the challenges, several groups have managed to move a step closer towards commercialising their approaches. One such example is Mintek (www.mintek.co.za), an established company with vast experience in bioleaching of gold from ores. [42] In partnership with BacTech, the company's commercial refractory gold bioleaching technology, Bacox^TM^, is currently applied at the Beaconsfield Gold Mine in Tasmania and the BioGold toll treatment facility located near the city of Laizhou, Shandong province, PR China. These facilities process between 70 and 100 tonnes of refractory gold concentrates a day and the bioleaching take place in large aerated stirred‐tank reactors where thermophilic iron‐oxidising bacteria are mixed with the refractory gold concentrates to leach out the gold. [43] Riding on its success in the use of biotechnology for the extraction of gold from ores, the company is now working on developing and optimising processes for the treatment of e‐waste.

Another example is Mint Innovation (www.mint.bio), a biometallurgy company based in Auckland, New Zealand. To recycle e‐waste, the team behind Mint Innovation combined both chemical and biological remediation steps in their workflow [44]. Grounded e‐waste is first mixed with acids and oxidants to leach out the copper, tin, and other less valuable metals, leaving behind precious metals such as gold and palladium in the solid remains. The solid e‐waste then undergoes another round of acid/oxidants leaching to obtain gold ions, which are subsequently exposed to the bacterium *Cupriavidus metallidurans* to absorb the gold ions and recover the precious metal. The company has been able to process around 1 metric ton of circuit boards in a week in their demonstration plant, recovering about 150 g of gold in the process. Mint Innovation is in the midst of building a commercial plant that is set to improve the throughput to 10 metric tonnes of circuit boards per day.

In 2018, the IT asset lifecycle management company N2S (www.n2s.co.uk) collaborated with research groups from Coventry University, UK to develop a pilot bioleaching plant to process e‐waste. They have demonstrated, for the first time, the direct application of electrowinning to PCBs bioleachate for the recovery of copper. [45] Shredded PCBs were introduced to the bacteria *A. ferrooxidans* after their growth phase and the electrowinning process was then carried out once the bioleachate reached the optimum condition for copper metal recovery. Characterisation of the recovered copper foil revealed a high purity (>99%), demonstrating the selectivity of the recovery process. This approach of copper separation from e‐waste could potentially serve as a pre‐treatment method to improve the recovery of precious metals from e‐waste.

## 4 SYNTHETIC BIOLOGY: A PROMISING TOOL IN ADVANCING BIOHYDROMETALLURGY

The use of microorganisms for e‐waste management opens up avenues for investigation using synthetic biology. Through the application of engineering principles to molecular biology, synthetic biology offers the tools to improve enzyme catalytic abilities, modify substrate specificities and raise toxicity tolerance, potentially accelerating the evolution of novel or enhanced capabilities needed to advance bioremediation and resource recovery. [46, 47] Shortcomings in microbial remediation have been the impetus in driving vast research into the development of genetically engineered microorganisms for bioremediative applications targeting all mechanisms—detection, adsorption‐chelation, absorption, and bioconversion— of metal capture and removal as extensively reviewed by Diep [48] and Sharma et al. [49].

Of these, the bulk of the studies related to metal accumulation and recovery remains focussed on using biologically engineered microorganisms to elucidate the molecular mechanisms behind their metal tolerance, oxidation, or degradation abilities, such as the discovery of delftibactin, a siderophore‐like non‐ribosomal peptide from *Delftia acidovorans* that can detoxify soluble gold, offering a mechanism for gold biomineralisation. [50] Several interesting studies, however, rise to the fore for their strong relevance in the potential application for bioleaching of e‐waste. In one study, *Pseudomonas putida* 06909, with characterised recombineering tools, was engineered to produce a high Cd‐affinity metal binding peptide EC20 that not only improved cadmium binding but also alleviated the cellular toxicity of cadmium. [51] In another study using an in silico genome‐scale metabolic model (GEM)‐based approach, gene targets that redirect the metabolic flux towards the production of cyanide were identified, leading to overproduction of the bioleaching agent and a concomitant improvement in gold and copper recovery. [52] Employing both genetic and chemical engineering, Zhu et al. constructed novel biotic‐abiotic coassemblies, comprising synthetic bacterial cells displaying the heavy‐metal‐capturing SynHMB on the cell surface and magnetic nanoparticles, which could remove cadmium and lead at high efficiencies. [53].

While an ever‐expanding range of toolkits has become available for a variety of microbial chassis, molecular tools for non‐model organisms, such as the bioleaching bacteria and archaea, are notably lacking. For example, despite their preeminent role in the biomining industry, *Acidothiobacillus* sp. and *Sulfolobus* sp. have rudimentary genetic tools for molecular manipulation. [35] Initial efforts in engineering these microorganisms for improved performance in bioleaching show promise. The expression of arsenic resistance genes in *A. ferrooxidans* generated strains with increased tolerance to arsenic while expression of the mer operon from *A. ferrooxidans* resulted in Mercury resistance in mercury‐ion sensitive variants. [54, 55] Although still far lagging than their model counterparts, importation of established toolkits, such as the shuttle vectors and genetic regulatory elements from *E. coli* into these acidophiles, serve as a starting point for establishing platforms for future work on metabolic or microbial engineering of biomining microorganisms for desired traits and, ultimately, improved bioleaching efficiencies. [56].

Another non‐model microorganism of interest is the cyanogenic bacteria *Chromobacterium violaceum* found to be one of the most effective microorganisms for the bio‐dissolution of gold. [57] Cyanogenic bacteria produces cyanide lixiviant, which complexes with gold facilitating the bioleaching process. The cyanide lixiviant is generated from the secondary metabolite hydrogen cyanide (HCN) produced by oxidative decarboxylation of glycine in a reaction catalysed by the enzyme (HCN) synthase encoded by the hcnABC operon. Regulation of this operon under quorum control limits the production of cyanide to 20 mg/L at the onset of the stationary phase. [58] To circumvent the limited cyanogenic capability of wild‐type C. *violaceum*, lixiviant production was decoupled from quorum control through the use of exogenous promoters (pBAD and pTAC) resulting in about 70% more cyanide production in the engineered strains and more than twice the recovery of gold. [59] An extension of the study introduced an additional copy of the cyanide producing operon to produce higher cyanide lixiviants was shown to boost gold recovery from 11% to 30%. [60] The cyanogenic capabilities of *C. violaceum* and its promising role in the targeted application of gold biorecovery from e‐waste makes it an attractive biological chassis for synthetic biology. Comparative proteomics analyses suggest that further increase in cyanogenesis is possible through lixiviant metabolic engineering and hence, with this in mind, a systematic genetic toolkit was developed to facilitate future engineering for biotechnological applications. [61].

While laboratory scale research provides insight into the tremendous potential of microorganisms in remediation and resource recovery, bold claims on their efficiency or cost‐effectiveness remain premature if the studies are not progressively scaled up to bioreactor configurations and onward to pilot scale setups and field tests. For the successful translation to industrial‐scale bioleaching processes, there is clearly a need to implement scale‐ups and validation tests, which could reveal bottlenecks such as decreasing bioleaching efficacy with increasing pulp density, pH fluctuations that affect the growth and activity of the acidophilic microorganism, or toxicity of the non‐metallic fraction of the WEEE, to name a few.

## 5 SYNTHETIC BIOLOGY: A POTENTIALITY FOR PLASTIC BIODEGRADATION

A greater part of the focus on e‐waste recycling lies in the recovery of precious metals, although not to be disregarded are the bulky plastics that are comparably valuable (Figure 2). [9] Plastics account for almost 37% of WEEE, but there are formidable hurdles to the recycling of e‐waste plastics mainly due to the mixed material composition and recalcitrant properties of synthetic polymers. [62] The constituents of e‐waste plastics include some 15 different polymer types with the typical composition comprising high impact polystyrene (HIPS, 27%), acrylonitrile butadiene styrene (ABS, 24%), polycarbonate (PC, PC‐ABS, 7%), polypropylene (PP, 5%) and polyethylene (PE, 2%), and the remaining consisting of polymer blends and other thermoplastics. [63] Compounding the problem is the presence of harmful additives such as BFRs and POPs. [64].

Even though established industrial processes for plastic recycling exist, the biological degradation of plastics could offer an ecologically friendlier strategy. The capability to degrade plastics has been reported in several microorganisms as extensively reviewed by Ru et al. [65] and Sameh et al. [66]. Isolated from a variety of environments, including soil, marine waters, landfill sites and waste sludge, these microorganisms have shown to have the ability to degrade synthetic polymers, such as PP, PE, polystyrene (PS), polyvinyl chloride, polyurethane (PU) and polyethylene terephthalate (PET), some even down to the respective simple monomeric units. [67] The degradation is based on the structural similarity of these plastics with natural polymers such as lignin present in the environment. Depolymerisation of synthetic plastics often involves microbial enzymes that target oxidisable C–C bonds, albeit with much lower efficiency. These naturally occurring enzymes include peroxidases, laccases, cutinases, esterases, hydrolases and lipases as detailed in Amobonye et al. [68] and Othman et al. [69].

To date, PET degradation by thermophilic actinobacteria via two key enzymes PETase and MHET hydrolase is arguably the most efficient among the currently known plastic degradation processes. [70] PET, however, is not a constituent of the e‐waste plastic stream. Hence, to strive towards a biological approach in e‐waste plastic recycling, it is imperative that the search and isolation persist for novel enzymes active on recalcitrant polymers relevant to e‐waste. [71] This is particularly pressing in the face of a dearth of reports on microbial biodegradation of high‐strength polymers ABS and HIPS, which constitute about 50% of e‐waste plastics. [72, 73].

Biodegradation of plastics is a very slow process under normal conditions. To overcome inherent limitations, the use of biological engineering would thus be pivotal in improving the efficiency of polymer degradation. As exemplified by the enhancement of PET hydrolytic activity by rational design, protein engineering efforts can be directed to the development of modified enzymes with the aim of improving specific binding capacities and catalytic activities towards a broader range of synthetic polymers. [74, 75, 76] This is particularly crucial when dealing with plastic material heterogeneity in e‐waste. The often time consuming process of molecular modifications towards gain‐of‐function or activity augmentation can be bolstered by maturing synthetic biology approaches such as whole‐genome sequencing, multi‐omics, automated high‐throughput mutant screening and novel strain construction using genome‐editing tools. [77] The growing availability of bioinformatics resources and computational biology tools can also aid in the advancement of plastic biodegradation by facilitating in silico research such as computer‐aided targeted mutagenesis and model‐based predictions of mechanistic enzyme‐polymer binding. [78].

**FIGURE 3:**
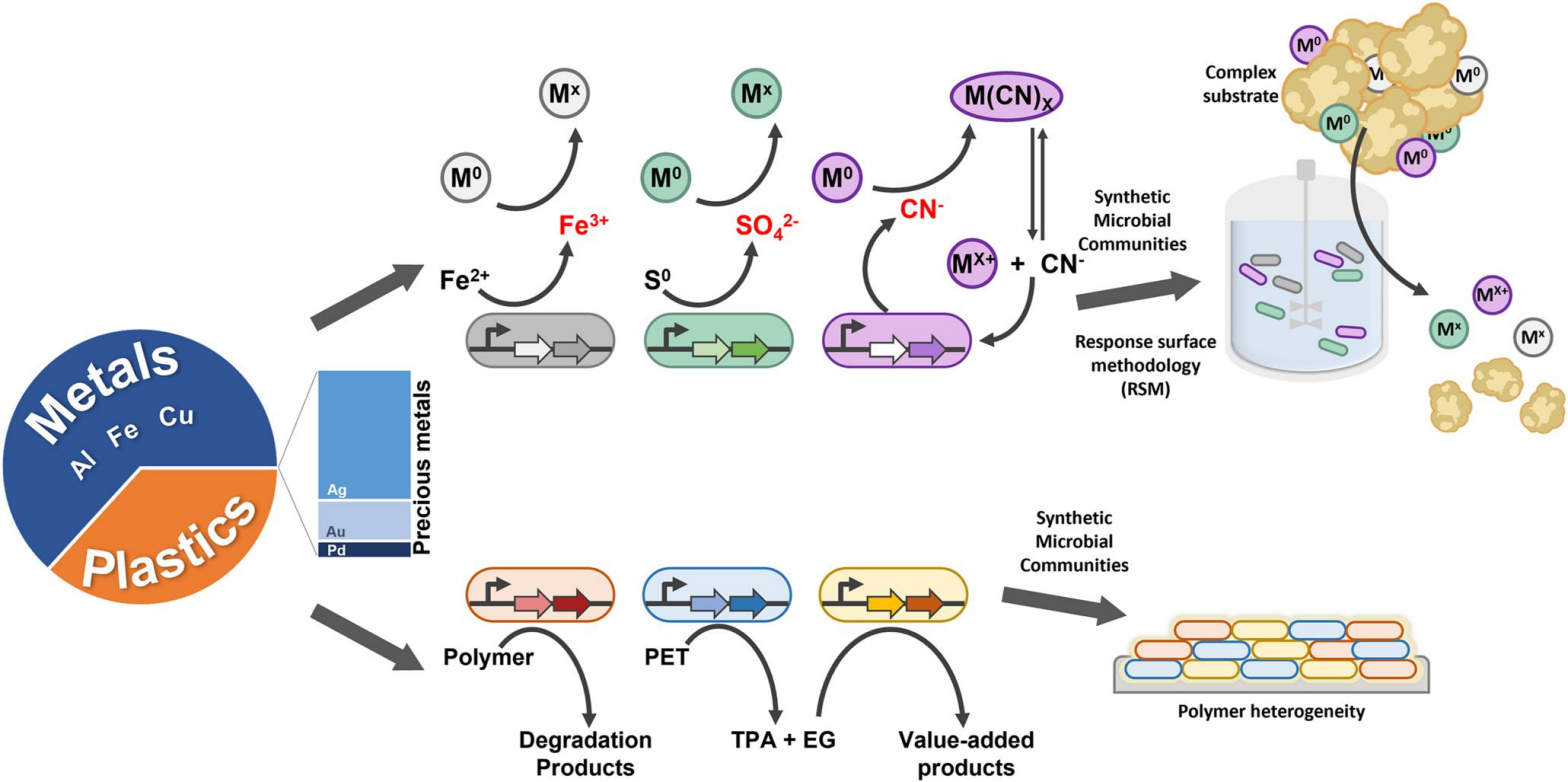
Biologically engineered microbes for the bioremediation of e‐waste. In 2016, the proportion of raw materials in e‐waste worldwide comprised base metals (aluminium, iron, copper) and precious metals (silver, gold, and palladium), and plastics. [9] Synthetic biology can be used to promote metal recovery by enhancing metal tolerance or increasing bioleaching efficiencies in iron‐ or sulphur‐oxidising microorganisms, or improving lixiviants bioproduction in cyanogenic bacteria. As more novel plastic‐degrading enzymes are uncovered, microorganisms can also be engineered to acquire plastic degradation abilities. In addition, metabolic engineering could facilitate the potential use of polymers as a feedstock in biotransformation for plastic upcycling. Future directions will be directed towards the use of mixed cultures or microbial consortia to tap on diversification of the function for greater efficacy against substrate heterogeneity. The prospective use of biofilm‐forming microbial chassis could also boost the colonisation and degradation of polymers. Besides biotic enhancements, a multipronged approach to maximise resource recovery in e‐waste can involve the application of response surface methodology (RSM) to determine optimal bioleaching parameters. M represents metal ions (M^x^) or its reduced form (M^0^)

The use of biologically engineered microorganisms has been the traditional strategy for achieving consolidated bioprocessing (CBP) by integrating all bioconversion reactions in a one‐step biological process. [79] In the wake of the huge challenges faced in designing and optimising all desired functional genes in one strain even with the advances in synthetic biology toolkits, there is growing recognition, particularly in e‐waste recycling, that engineering of mixed cultures and even microbial consortia to compartmentalise complex tasks and functionalities would be the way forward (Figure 3). [80] Notably, the use of tailored microbial consortia has shown higher biodegradation efficiencies of PE, PU, and PET compared to individual strains. [77, 78] With complex microbial communities, it is postulated that different microorganisms may exploit metabolic cross‐feeding and express distinct degrading enzymes when cultured with the different plastic materials, and that combinations of selected bacteria may have synergistic effects on biodegradation.

A remarkable characteristic of the PET‐degrading bacterium *Ideonella sakaiensis* is its innate ability to utilise the degradation products as a major carbon and energy source for growth. [70] Tapping on the microorganism's ability to assimilate PET degradation products as building blocks for growth opens the possibility of plastic waste as a novel feedstock for microbial biotransformations. [81] Challenged by the low cost of virgin plastics, research efforts have been directed away from recycling the recovered monomers for bio‐plastics production but have instead pivoted towards engineering metabolic pathways in microbes to bio‐upcycle synthetic polymers to produce value‐added products, such as high‐value aromatics and innovative chemicals for novel materials. [65] Riding on this approach is the EU Horizon 2020 research project MIX‐UP—Mixed plastics biodegradation and upcycling using microbial communities, which ambitions to explore novel sustainable routes to valorise plastic waste streams. [82] The project aims to work towards controlled enzymatic and microbial degradation of pre‐treated plastic waste using heavily engineered, plastic type‐specific enzyme blends, and subsequent microbial conversion to polymers and value‐added chemicals by self‐sustaining microbiomes.

## 6 CHALLENGES IN INDUSTRIAL IMPLEMENTATION

The utilisation of microbes for the remediation of e‐waste provides a greener, less expensive, and less toxic avenue for e‐waste recycling. However, there are several limitations that need to be addressed before this process can be implemented on an industrial scale (Table 1). [22, 83].

**TABLE 1:** Advantages and disadvantages of using microbes for bioremediation of electronic waste. [22, 83]

Advantages	Disadvantages
1. Simple and inexpensive	1. Bioremediation activity can be easily affected by microbes' tolerance towards toxic e‐waste components
2. Eco‐friendly technique	2. Bioremediation process is usually slower compared to remediation through physical and chemical processes.
3. Avoid the use of harsh chemicals, high temperature, and pressure	3. Limited availability of molecular toolkit to improve the rate of bioremediation and storage of microbes
4. Permanently eliminate contaminants through biochemical transformation or mineralisation	4. Scaling up of the bioremediation process is complex and time‐consuming

### 6.1 Toxicity of organic and inorganic components in e‐waste

E‐waste contains diverse materials, including hazardous organic substances such as PCBs, polybrominated biphenyls, polybrominated diphenyl ethers, and BFRs, and inorganic metals such as lead, Mercury, chromium, and cadmium. [5, 84, 85, 86, 87] Concentrations of these components are likely to differ from batch to batch depending on the myriad types of e‐waste. This contrasts with most laboratory‐based studies where the bioremediation activity of the microbes is usually optimised using a uniform source of e‐waste, and the microbes' tolerance towards the hazardous components is well‐defined. As a result, transiting from lab‐scale to industrial‐scale bioremediation poses a huge challenge for consistency in bioremediation efficiency as the viability of microbes, and consequently their activities, are likely to vary from batch to batch, thus limiting their potential for application in commercial scale e‐waste recycling.

### 6.2 Slow process in comparison to physical and chemical remediation

Even though bioremediation is an effective and less hazardous method for e‐waste recycling, its sluggish pace makes the process a weak contender as compared to the use of physical and chemical means to recycle e‐waste. To gain ground, the rate of bioremediation needs to be drastically improved, and this often requires an in‐depth study on the mechanism of the bioremediation process as well as the genetic engineering of microbes for better performance. In addition, the composition of e‐waste can affect the speed of the bioremediation process, with reports demonstrating inhibitory effects on bioleaching by competing metals in the e‐waste. [20, 22]. This inhibition could make the bioleaching of PCBs 20 times slower than its potential rate.

### 6.3 Limited availability of molecular toolkits

Unlike common microbes such as *E. coli, Saccharomyces cerevisiae,* and *Pichia pastoris*, microbes that display great potential for e‐waste bioremediation (*Chromobacterium violaceum, Acidithiobacillus ferooxidans*, thermophilic bacteria, acidophilic bacteria) often have very limited molecular toolkits available for genetic manipulation. [35] This makes the engineering of these species to enhance their bioremediation activities difficult and slow. Besides the lack of molecular toolkits, preservation of these microbes is also a challenge as some of these microbes exhibit poor viability following preservation. [35, 88] Differences in the microbes' cell structure and their overall sensitivity towards ultra‐low temperatures and desiccation processes often result in failure of preservation using standard methods as observed in acidophilic thermophilic microorganisms. [89, 90] To circumvent the problem, these microbes are maintained as live cultures requiring continual subculturing and storage at temperatures below optimal growth requirements, and with slow release substrates to prolong viability. [91] Hence, with the push to discover novel microorganisms for bioremediation, tandem efforts must be made to develop methods for the perseveration of these microbes for ease of use.

### 6.4 Difficulty in scaling up of the bioremediation process

Moving an idea from laboratory‐scale success to a pilot‐scale demonstration plant and onwards to an economically profitable commercial scale is a road fraught with challenges. While physical–chemical technologies can mostly be scaled up with relatively high predictive reliability, biological applications, on the other hand, are more problematic in which how well a remediation treatment will work and how long that treatment will require is plagued with uncertainty as a result of the use of live organisms. It has been reported that in bioremediation scale‐ups, laboratory‐scale kinetics can exceed field‐scale kinetics and the duration of treatment under‐predicted in excess of 100% and by as much as 11,900%. [92] Aside from the complex, capricious nature of microorganisms, biological treatment processes are also sensitive towards the physical (e.g. temperature, oxygen level) and chemical (e.g. pH, humidity, nutrition) conditions of the reaction setup, and these conditions are often affected when translating from laboratory‐scale systems to field‐scale designs. [93] This, in addition to the introduction of spatial heterogeneities, and additional mass transport mechanisms and limitations, render the engineering of bioremediation industrial scale‐ups an arduous task.

## 7 THE ROAD AHEAD: THE FUTURE OF E‐WASTE BIOREMEDIATION

**FIGURE 4:**
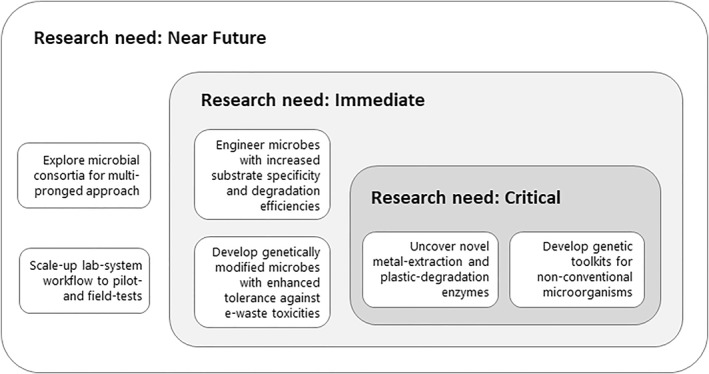
Roadmap to addressing the research needs to overcome technological gaps in the advancement of bioremediation of e‐waste using biologically modified microorganisms

Despite being cumbered by limitations that are not trivial, commercial implementation of e‐waste bioremediation and resource recovery remains highly desired goals in a global drive towards a zero‐waste circular economy. To advance the biological approach to e‐waste management, gaps or inadequacies in our current knowledge or methods have to be identified and addressed by directing rigorous research efforts to overcome technological hurdles (Figure 4).

One of the utmost research needs is the expansion of the biological arsenal capable of driving the enzymatic remediation of e‐waste, particularly in the area of plastic degradation where enzymes specific for diverse polymers are sorely lacking. As exemplified by the uncovering of more than 500 potential PET hydrolases globally distributed in marine and terrestrial metagenomes, and the anomalous discovery of PR‐degrading bacteria in insect guts, efforts to prospect from microbial communities in natural environmental niches or from indigenous microbes inhabiting polluted sites or operation facilities should continue. [94] Here, automation provides a means to perform the tedious task of conducting high throughput screening of huge gene pools for desirable novel functions. Automation, however, requires substantial capital investment on specialised facilities and skills and a possible workaround will be for companies and the scientific community to collaborate with biofoundries equipped with automation systems and high‐throughput analytical equipment to accelerate the screening process. [95].

Indisputably, the most pressing issue that needs to be resolved is the efficiency, and consequently speed, of the bioremediation process. Two aspects, namely enzymatic performance and toxicity tolerance, are important determinants affecting bioremediation efficiency. The way forward to achieving significant improvements in enzymatic performance will likely rely on the use of biologically modified microbes. The Chinese–European MIX‐UP project, for example, endeavours to create a novel workflow to recycle plastic waste through the use of heavily engineered enzyme cocktails. [82] Through the application of the ‘design, build, test, and learn’ strategy in the engineering of the microbes, synthetic biology can enable a systematic and iterative construction of the engineered microbes and feedback loop analyses. [47] To facilitate genetic engineering, toolkits containing standardised genetic parts for building transcriptional units and operons are needed to generate mutant libraries in vitro for the selection of desirable phenotypes. [96, 97, 98, 99] For this purpose, better efforts will be needed to develop molecular toolkits for the non‐conventional microbes naturally adept with niche capabilities in bioremediation. This can be done by tapping on the established BioBrick system for model organisms in the bioremediation of gold, cobalt, nickel, and Mercury. [100, 101, 102] Biofoundries can again play a vital role in screening exhaustive mutant libraries. For instance, Allonnia, a spin‐off venture of the leading biotech company Ginkgo Bioworks, has plans to engineer microbes through synthetic biology to remediate organic pollutants in waste. Maturing fields in computer‐assisted protein engineering and droplet‐based microfluidics can also enable improvements in directed evolution of enzymes of interest, potentially hastening research efforts in targeted improvements of bioremediation capabilities of modified microbes. [103].

Concomitant with growing research studies is the realisation that dependence on a singular biological workhorse, even if highly engineered, to remediate complex e‐waste is a tall order. Research directions have thus been re‐modelled to include the exploitation of microbial communities to better address the material heterogeneities of e‐waste, whether metals or plastics. While holding much potential for dealing with the complex compositions of e‐waste, mixed cultures are, in reality, difficult to implement as maintaining favourable growth conditions for different microbes and ensuring the cellular environment optimal for different enzymes are daunting challenges. Equally important is the need for the process scale‐up from laboratory to pilot and field operations. This is a crucial step in the progress from proof‐of concepts to industrially applicable technologies. Consideration of the above issues in the early design stages would be useful in the path to developing a commercial bioremediation process.

Besides technical challenges, complementary e‐waste logistics and management are also critical gaps to close in the bid towards eventual commercialisation of bioremediation of e‐waste. For instance, segregating e‐waste based on their composition can help with material homogeneity, thereby alleviating the issue of bioremediation activity inconsistency caused by interfering components in the e‐waste. In addition, the inclusion of a pre‐treatment step before introducing the e‐waste to the microbes could also help to improve the rate of biodegradation. [104] Another possible workflow will be the use of a two‐step bioleaching approach, such as that demonstrated by Shah et. al. where the biomass was allowed to grow in the first step prior to the addition of PCBs, resulting in a 2–3.12 fold increase in copper, zinc, and nickel extraction to recover copper, zinc, and nickel from PCBs. [105].

Finally, the following concerns ought to be deliberated for future recycling technological innovations: [1] In the drive to achieve optimal bioremediation efficacies and greater resource recovery rates, the bioremediation workflow should be sustainable in terms of cost and ecological footprints; [2] To gear towards accessible commercialisation, the technology involved should be suitable for adoption by small and medium‐sized enterprises in terms of investment or infrastructure. The operational technicalities and costs should also be put forward through the development of pilot scale tests by the scientific community; [3] Due to the complex nature of e‐waste, a single bioremediation technology is unlikely to be sufficient to accomplish complete e‐waste recycling. Therefore, stakeholders should remain open to integrating various recycling technologies, including physicochemical treatments, for the complete remediation of e‐waste. [15].

## 8 CONCLUDING REMARKS

The use of biologically engineered microbes for bioremediation of e‐waste, although promising, is still in its infancy and there remains a lot more research and development to be committed before the technology can be adopted commercially. Progress has been made to improve cell tolerance against e‐waste toxicity, to tackle the need to recover multiple metals in a single process, and to increase the recovery efficiencies of bioleaching. However, studies on scaling up these laboratory scale successes are lacking in the scientific field, restricting the commercial implementation of e‐waste bioremediation. Therefore, research communities and industries should work together to allow a smooth transition from the laboratory scale bioremediation process to the field operational scale. In addition, the limited availability of molecular toolkits for genetic engineering of uncommon microbes that display great potential towards e‐waste bioremediation is slowing down the process of improving their metal recovery efficiencies. Through the application of the ‘design, build, test, and learn’ strategy in synthetic biology and automation, these much‐needed molecular toolkits can be developed at a faster pace. Proper e‐waste management and planning of the bioremediation workflow are also critical gaps to close in the progress towards eventual commercialisation of the bioremediation of e‐waste. Once these technical hurdles are crossed, it is important to deliberate whether the technology requires integration with other secondary or even tertiary recycling processes for complete e‐waste remediation, and if the technological workflow is ecologically and economically sustainable and suitable for decentralised adoption by small and medium‐sized enterprises in terms of investment or infrastructure. Although premature to count as success stories, nascent industry players, such as Mint Innovation and N2S, have taken inceptive steps towards deploying microbes in their e‐waste recycling workflow and these forerunners may usher in the establishment of commercial‐scale bioremediation of e‐waste in the foreseeable future.

## CONFLICT OF INTEREST

The authors declare no conflict of interest.

## Data Availability

Data sharing is not applicable to this article as no new data were created or analysed in this study.
