# Corrigendum: Biologically engineered microbes for bioremediation of electronic waste: Wayposts, challenges and future directions

**DOI:** 10.1049/enb2.12023

**Published:** 2022-08-23

In Ref. [1], the authors wish to acknowledge the funder. Below is the acknowledgment statement:

“This project is partially supported by the National Research Foundation, Singapore, under its Competitive Research Programme (Award # NRF‐CRP22‐2019‐0005)”.
